# Vulnerability of Polar Oceans to Anthropogenic Acidification: Comparison of Arctic and Antarctic Seasonal Cycles

**DOI:** 10.1038/srep02339

**Published:** 2013-08-01

**Authors:** E. H. Shadwick, T. W. Trull, H. Thomas, J. A. E. Gibson

**Affiliations:** 1 Antarctic Climate & Ecosystems Cooperative Research Centre, University of Tasmania, Hobart, Tasmania, Australia; 2 Institute for Marine and Antarctic Studies, University of Tasmania, Hobart, Tasmania, Australia; 3 CSIRO Marine and Atmospheric Research, Hobart, Tasmania, Australia; 4 Department of Oceanography, Dalhousie University, Halifax, Nova Scotia, Canada

## Abstract

Polar oceans are chemically sensitive to anthropogenic acidification due to their relatively low alkalinity and correspondingly weak carbonate buffering capacity. Here, we compare unique CO_2_ system observations covering complete annual cycles at an Arctic (Amundsen Gulf) and Antarctic site (Prydz Bay). The Arctic site experiences greater seasonal warming (10 vs 3°C), and freshening (3 vs 2), has lower alkalinity (2220 vs 2320 μmol/kg), and lower summer pH (8.15 vs 8.5), than the Antarctic site. Despite a larger uptake of inorganic carbon by summer photosynthesis, the Arctic carbon system exhibits smaller seasonal changes than the more alkaline Antarctic system. In addition, the excess surface nutrients in the Antarctic may allow mitigation of acidification, via CO_2_ removal by enhanced summer production driven by iron inputs from glacial and sea-ice melting. These differences suggest that the Arctic system is more vulnerable to anthropogenic change due to lower alkalinity, enhanced warming, and nutrient limitation.

The polar oceans are sensitive to increasing global temperature and increasing concentrations of atmospheric carbon dioxide (CO_2_) (refs. 1, 2), with the impacts of climate change expected to be particularly large in ice-covered regions. Compared to other oceans, the Arctic and Southern Oceans remain under-studied at the annual scale, with the majority of observations restricted to the ice-free summer and autumn seasons^3^,^4^. In recent decades the rapid loss of sea-ice from the Arctic Ocean has exceeded even the most pessimistic model projections^5^,^6^. The Southern Ocean has exhibited significant and regionally variable changes; satellite observations indicate that the Amundsen Sea has undergone both warming and a decrease in the extent of summer sea-ice^7^. In contrast, the Ross Sea has undergone increases in both the extent of sea-ice and the duration of ice cover^8^.

Changes to the polar environment affect ocean acidification via both the chemical buffering capacity and the modulation of biological carbon uptake (through changes to light and nutrient availability). To explore how these changes are likely to differ in the circumpolar Southern Ocean versus the largely landlocked Arctic Ocean, we compare two uniquely high-resolution observational data sets of complete annual cycles in the Arctic and Antarctic, on the heels of the recent 3rd International Polar Year (IPY) - a collaborative effort to study the Polar regions. These records are novel in that they cover a full annual cycle of the CO_2_ system in both the Arctic^9^ and Southern Ocean^10^. The observations were collected ~15 years earlier in the Antarctic, so that temporal differences could in principle affect the comparison. Because changes in East Antarctica surface temperatures^11^ and sea-ice extent^11^,^12^ over this period appear to have been minimal, we assume that climate warming over this period can be neglected, and to assess the change in anthropogenic CO_2_ loading, we projected Antarctic observations forward to 2007, (when the Arctic data were collected), with the assumption that Southern Ocean surface waters approximately track the increase in atmospheric CO_2_. This assumption has been validated not only for the Southern Ocean, via broad scale observations^13^, but at long-term CO_2_ time series stations at both Bermuda^14^ and Hawaii^15^. Furthermore, new observations from Prydz Bay in 2010/11 indicate increases in inorganic carbon that are consistent with the observed atmospheric increase between 1994 and 2010^16^ (see Methods for a detailed description of the forward projection of the Antarctic data to the year 2007).

While whole regions cannot be adequately characterized by observations made at two locations, comparison of the carbon cycle in these two ice-covered systems lends insight into the drivers of seasonal changes, and into the differing responses of the carbonate system to projected future perturbations. Moreover, the Amundsen Gulf site is broadly representative of coastal waters in the Western Arctic^17^ and throughout the Arctic Archipelago^18^, while Prydz Bay is broadly representative of coastal conditions in the Ross^20^,^21^ and Weddell Seas^22^ in the Antarctic, in terms of water properties important to the carbonate system, shown by comparison of summer surface properties in several other locations^9^,^10^,^17^,^18^,^19^,^20^,^21^,^22^,^23^ (see Tables 1 and 2). However, we note that the Amundsen Gulf system is not representative of areas of the Arctic Ocean under the influence of Atlantic inflow, notably the Barents Sea^19^ (see Table 1), and the Prydz Bay system is significantly different from the warmer, fresher, and more rapidly changing Antarctic Peninsula^23^ (Table 2). From our assessment of the differing response of these two systems, the larger seasonal temperature cycle in the Arctic, and the greater nutrient availability in the Antarctic emerge as key variables. This examination also illuminates the importance of collecting data covering full annual cycles in the Polar regions in order to develop baseline assessments against which long-term trends and changes can be detected.

## Results

### Amundsen Gulf

**Figure 1:**
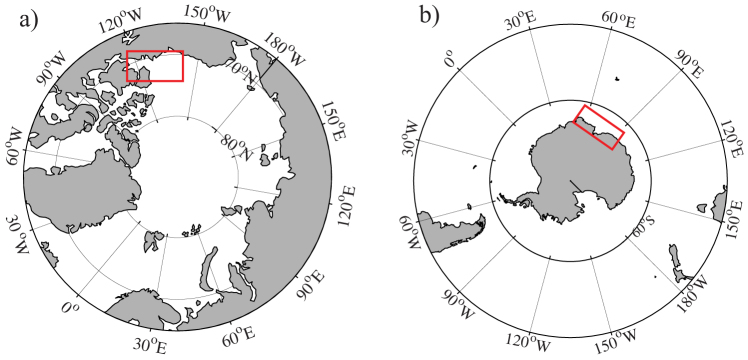
Locations of the study areas in (a) Amundsen Gulf, Canadian Arctic Archipelago, and (b) Prydz Bay, East Antarctica. Figures were drawn using MatLab.

The Amundsen Gulf (AG) is part of a large channel adjacent a narrow continental shelf, connecting the southeastern Beaufort Sea to the Canadian Arctic Archipelago (Fig. 1a). The water column structure in the region can be simplistically described as a three-layer system: the relatively fresh Polar Mixed Layer (PML, salinity 29–31, 0–50 m depth), the Pacific halocline (salinity of 31–33, 50–200 m depth), and deep waters of Atlantic origin (salinity 34.4–34.8 > 220 m)^9^. The circulation in Amundsen Gulf is dominated by a subsurface (below 50 m) flow toward the Beaufort Sea, there is a weaker surface circulation bringing water into the region from the West^9^. The seasonal thermal cycle is very large, with surface waters warming by roughly 10°C. A recurrent physical feature in Amundsen Gulf is the Cape Bathurst polynya, which opens in mid-to-late June and remains open well into October, although with significant interannual variability in extent and timing^24^. The year 2007 is associated with anomalously low September sea-ice extent in the Arctic, though this record has now been surpassed in 2012 with similar timing of the onset of seasonal melt^25^. Early sea-ice melt may cause an early onset of open water phytoplankton production, since interannual variability in the neighbouring Cape Bathurst Polynya has been shown to correlate with the timing of seasonal ice melt^24^. Annual primary production in the region has been inferred from remote sensing with values ranging from 90 to 175 g C m^−2^ yr^−1^ over the period from 1998 to 2004^24^. The region acts as a moderate sink for atmospheric CO_2_ with annual uptake of roughly 1 mol C m^−2^ yr^−1^ (ref. 9).

### Prydz Bay

Prydz Bay (PB) is the largest embayment on the broad East Antarctic continental shelf, lying offshore from Australia's Davis Station in the Vestfold Hills (Fig. 1b). The sea-ice breaks up in late December to early January and the region remains ice-free until fast ice reforms in early March^10^. The main flow of water inshore is along the coast from the West Ice Shelf (Fig. 1b), keeping salinity in Prydz Bay close to open ocean levels, in the range from 33 to 34.5. The seasonal temperature cycle of surface waters is quite small at <2°C. Primary production estimates in Prydz Bay (from remote sensing observations over the period from 1997 to 2002) are similar to those observed in Amundsen Gulf, on the order of 100 g C m^−2^ yr^−1^ (ref. 26). The estimated annual uptake of atmospheric CO_2_ is on the order of 1 to 2 mol C m^−2^ yr^−1^, as a result of strong surface undersaturation in summer and the assumption that the sea-ice provides a barrier to outgassing during autumn and winter, when waters are supersaturated with respect to atmospheric CO_2_ (ref. 10).

### Annual cycles

**Figure 2:**
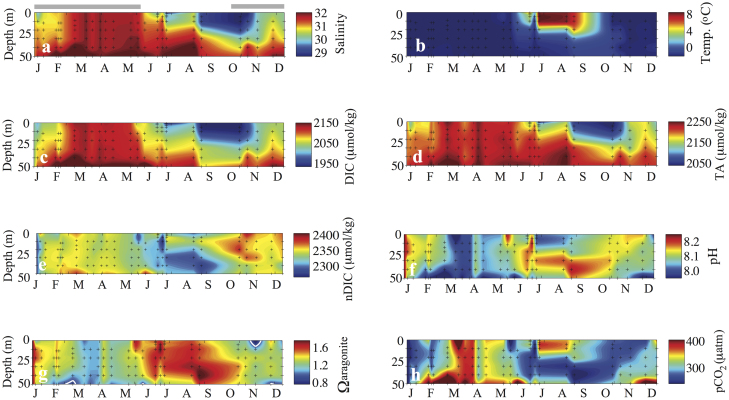
Annual cycles of a) salinity, b) temperature, c) DIC, d) TA, e) salinity-normalised DIC (nDIC), f) pH, g) Ω, and h) pCO_2_, in the upper 50 m, in Amundsen Gulf. The seasonal cycle of sea-ice is shown schematically above panel a). This figure is modified from Shadwick et al. (2011)^9^.

**Figure 3:**
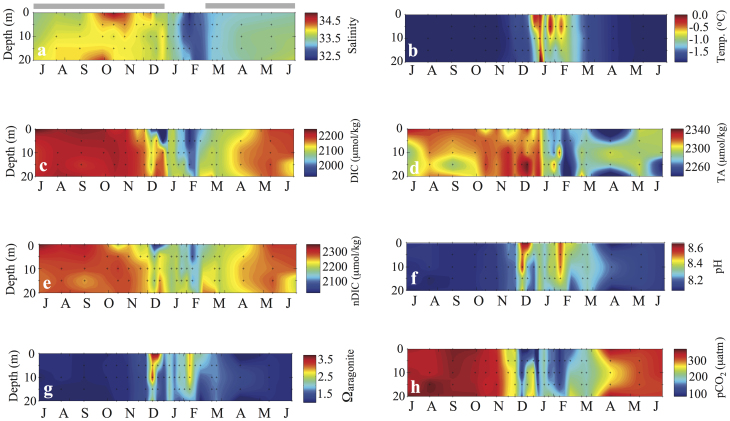
Panels as in Fig. 2 for Prydz Bay, East Antarctica (drawn from data in Gibson and Trull, 1999^10^).

The annual cycles of salinity and temperature (and the inorganic carbon system parameters) for Amundsen Gulf and Prydz Bay are shown in Figs. 2 and 3, respectively. The data from Prydz Bay have been plotted from July to June to allow comparison with the January to December annual cycle in Amundsen Gulf. The first thing to note is the lower salinity of the Arctic system, reaching a maximum of ~32, in comparison to the maximum of ~34.5 in the Antarctic. Differing seasonality accentuates this difference. At both sites, the salinity maximum (and temperature minimum) occurs well into winter, nearing the winter-to-spring transition, due to brine rejection from ongoing sea-ice formation. Spring then brings freshening, but this is larger in the Amundsen Gulf (decreasing salinity by ~3) than in Prydz Bay (decreasing salinity by ~2), and in further contrast to Prydz Bay, there are two pulses of freshwater input to the surface layer in Amundsen Gulf. The first in late-May is associated with the onset of riverine discharge, the second, coincident with the salinity minimum (S = 29) is observed several months later (in September) due to both sea-ice melt and river runoff. There are significant contrasts in temperature as well. While both sites exhibit cold weakly-stratified waters through winter, the intensity of the brief period of summer warming is greatly different. In Prydz Bay the water temperature rises from near freezing (−1.8°C) to a maximum of 0°C (Fig. 3b), while in Amundsen Gulf the waters warm to more than 8°C in summer (Fig. 2b). This summer warming also persists more than a month longer in the Arctic.

The importance of the formation/melting of sea-ice in controlling the seasonal changes in inorganic carbon system in Prydz Bay and Amundsen Gulf is apparent from the similarity among the annual evolutions of salinity, DIC, and TA. As with temperature and salinity, there are significant differences between the regions throughout the year as well as seasonally – Arctic waters have lower pH and carbonate saturation state as a result of their lower salinity and TA. In winter (DJF in Amundsen Gulf, and JJA in Prydz Bay) an increase in salinity due to brine rejection during sea-ice formation is coincident with an increase in DIC (and TA). Part of the increase in DIC in the autumn and winter period is due to respiratory remineralization of organic matter (Table 3), which is independent of changes in salinity and can be seen in the plot of salinity-normalised DIC (nDIC, Fig. 2e). In spring, roughly two months before sea-ice melt-out (March in Amundsen Gulf and November in Prydz Bay), a decrease in surface DIC (and nDIC) is observed in both regions (Figs. 2 and 3, panels c and e), due to the uptake of inorganic carbon by under-ice algae, that make a significant contribution to net community production (NCP, ~35% in AG, and ~15% in PB, see Table 3 and Methods). The open water period, longer in Amundsen Gulf, coincides with both salinity and DIC minima (Figs. 2 and 3). Biological production continues through August in Amundsen Gulf, despite the onset of nutrient limitation of growth rates in late June, and through February in Prydz Bay (Table 3).

Biological production is the main factor driving the seasonal cycles in pH and aragonite saturation state (Ω) in both regions (Fig. 2f and g, Fig. 3f and g). In the subsequent discussion, comparison of carbon system parameters is made using the Prydz Bay data projected to 2007, when the Amundsen Gulf observations were made. The maximum (summer) values of pH (~8.5) and Ω (~3.4) are significantly higher in Prydz Bay, and coincide with maximum NCP (Table 3). By contrast, the maximum pH in Amundsen Gulf (pH 8.15) occurs at the onset of ice-melt (late May, early June), and the peak summer values of Ω (1.6 to 1.8) occur in JJA, lagging the seasonal increase in pH. This occurs in Amundsen Gulf because surface warming causes a significant increase in pCO_2_ (on the order of 90 μatm see Fig. 2h), and corresponding decrease in pH, despite the ongoing carbon drawdown by photosynthesis. Thus there is a decoupling between the seasonal enhancement of pH and Ω due to the opposing influence of biology and thermodynamics on surface pH which results in maximum values of pH in spring (due to under-ice biological production, Fig. 2f and Table 3) and again in autumn (due primarily to surface water cooling, see also Fig. 2b), and a magnitude of seasonal change in pH of roughly 0.2 units. In contrast to pH, the maximum values of Ω do correspond with the height of the open water phytoplankton bloom in summer in Amundsen Gulf, just as they do in Prydz Bay, although the magnitude of the Amundsen pH and Ω cycles are notably smaller than those of Prydz Bay (0.2 vs 0.4 in pH, and 0.4 vs nearly 3.0 in Ω). The weak seasonal warming in Prydz Bay (~2°C, Fig. 3b) allows the smaller biological carbon drawdown of 2.4 mol C m^−2^, (compared to roughly 3.6 mol C m^−2^ in Amundsen Gulf from April to August, see Table 3), to drive a similar uptake of atmospheric CO_2_ (1 to 2 mol C m^−2^) despite a shorter open water season (see also refs. 9 and 10).

## Discussion

The Arctic system has a smaller capacity to buffer decreases in pH and Ω (aragonite) from anthropogenic CO_2_ uptake due to lower alkalinity, and strong seasonal warming that prevents summer primary production from significantly elevating pH and carbonate saturation. The Antarctic system is more strongly buffered by enhanced alkalinity (relative to the Arctic system) and exhibits a much smaller seasonal warming allowing large summer increases in both pH and Ω from primary production. These differences suggest that the carbon system response to anthropogenic CO_2_ forcing will be regionally specific. To investigate the differing sensitivities in the Arctic and Antarctic systems, we apply a surface water equilibration with an atmospheric CO_2_ increase equivalent to the IPCC B1 scenario^27^ with concentrations reaching ~550 ppm CO_2_ in 2100 (see Methods). We assume that the seasonal cycles remain unchanged, and apply a simple approach that includes warming and freshening, as well as the uptake of anthropogenic CO_2_. The resulting seasonal cycles of pH and Ω are thus not treated as projections of monthly or seasonal values of these parameters, instead the exercise yields insight into the sensitivity of the carbonate system to anthropogenic forcing, and importantly, the differing response in the Arctic and Antarctic to this forcing (see Methods for a full description of the forward projections and the caveats associated with this approach).

**Figure 4:**
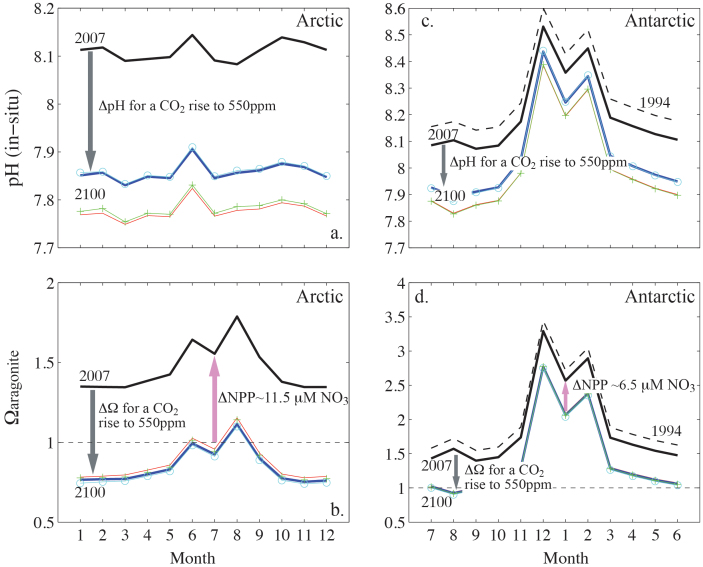
Estimated seasonal cycles in pH (top) and Ω (bottom) between the years 2007 and 2100 in Amundsen Gulf (left), and Prydz Bay (right, 1994 values given by the black dashed line). The 2007 values (solid black lines) are compared to 2100 with: anthropogenic CO_2_ increases only (solid blue lines, grey arrows); CO_2_ increase and warming (red line); CO_2_ increase and freshening (pale blue line with open circles); CO_2_ increase, warming, and freshening (green line with + symbols). The pink arrows indicate the required additional NPP, or NO_3_ uptake required to buffer the summer time decreases in pH and Ω due to anthropogenic CO_2_ uptake.

In Amundsen Gulf, the winter pH is reduced by uptake of anthropogenic CO_2_ from roughly 8.1 in 2010 to 7.85 in 2100, with further reduction to below 7.8 by associated warming of 6°C (Fig. 4a). Aaragonite saturation state decreases from winter values of roughly 1.4 in 2010 to undersaturated values of 0.8 in 2100 (Fig. 4b), with negligible additional (positive) changes from warming. Including freshening of 0.1 units does not significantly change projection of either pH or Ω. The summer values are also decreased, with maximum pH (in June) of roughly 7.9 in 2100 with seasonal warming (assumed unchanged) decreasing pH to near winter values. The seasonal increases in Ω results in values up to 1.2 in August, however, over the majority of the seasonal cycle, Ω is undersaturated in the Arctic system in the year 2100.

**Figure 5:**
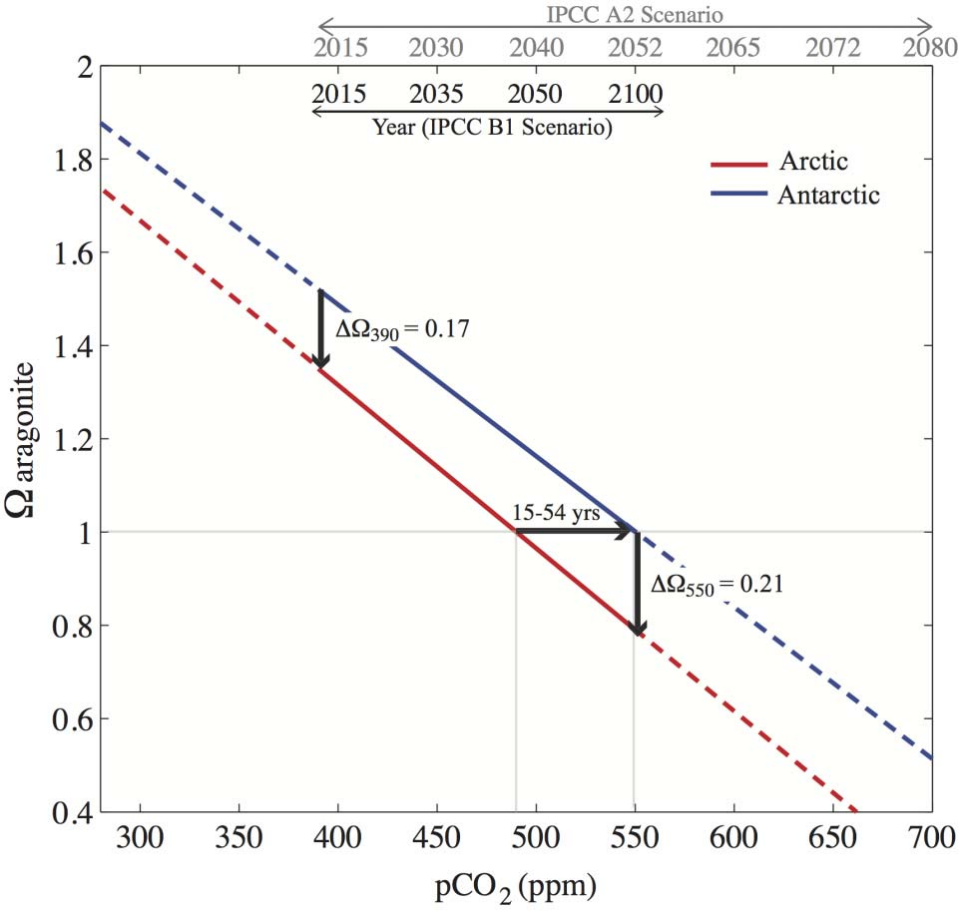
The relationship between (atmospheric) CO_2_ and Ω in Amundsen Gulf (red) and Prydz Bay (blue), and the timing (top horizontal axes) given by both the B1 and A2 IPCC SRES scenarios^27^. The more alkaline Antarctic waters may delay the onset of carbonate undersaturation by 15 (A2) to 54 (B1) years.

In Prydz Bay, winter values are decreased from pH of roughly 8.1 in 2010 to 7.9 in 2100, and Ω declines from 1.5 to roughly 1.0 (Fig. 4c and d). A different situation from that described above is observed in the summer season. The Prydz Bay system has a greater capacity to buffer anthropogenic CO_2_ addition due to the more alkaline waters. In summer, the pH is reduced from roughly 8.4 in 2010 to roughly 8.3 in 2100 (Fig. 4c). Similarly, Ω decreases from roughly 3.0 in 2010 to roughly 2.5 in 2100 (Fig. 4d). The impact of increased temperature results in a greater decline in both summer and winter pH, but the smaller projected temperature increase for the Antarctic (3°C) has a smaller impact on pH. These findings suggest that, of the two, the Arctic system may more vulnerable to anticipated future changes in ocean pH and Ω, due to the smaller range in the (present day) seasonal cycle, and the weaker capacity to buffer anthropogenic CO_2_ uptake; winter carbonate undersaturation may be reached in the Arctic system 15 to 54 years before it occurs in the Antarctic system (Fig. 5).

A greater capacity to buffer the CO_2_ system is also present in the Antarctic than the Arctic, because of the higher nutrient concentrations available in the Southern Ocean. We estimated the increase in net primary production (NPP), or nitrate (NO_3_) uptake that would be required to buffer the summer decreases Ω (and pH) due to anthropogenic CO_2_ uptake (Fig. 4). In the Amundsen Gulf system, an additional uptake of 11.5 μmol kg^−1^ of NO_3_ would be required to offset the decrease in pH and Ω between 2010 and 2100. However, the Arctic Ocean and continental shelves are predominantly nutrient limited^28^,^29^. In 2007, the winter (maximum) NO_3_ concentration in Amundsen Gulf was 16 μmol kg^−1^ at a depth greater than 60 meters. In early March, the surface concentration reached an annual maximum of roughly 10 μmol kg^−1^, and was rapidly reduced to less than 5 μmol kg^−1^ after the onset of under-ice biological production^30^. The surface waters became NO_3_ limited (NO_3_ < 1 μmol kg^−1^) in early June, two months before the cessation of biological production in the region^9^,^30^. Thus, despite suggestions that NPP in the Arctic may increase in the future, due to an extended growing season caused by earlier seasonal sea-ice melt, or the disappearance of sea-ice^31^, observations of nutrient availability in Amundsen Gulf indicate that increases in NPP of sufficient magnitude to mitigate anthropogenic CO_2_ uptake are unlikely.

In the more strongly buffered Prydz Bay system, an additional NPP of roughly half the amount estimated for Amundsen Gulf, 6.5 μmol kg^−1^ NO_3_, is required to overcome the projected summer decreases in pH and Ω (Fig. 4b). Productivity of the ‘high nutrient, low chlorophyll (HNLC)’ waters of the Southern Ocean are limited not by nitrate, but by iron (Fe)^32^,^33^. Observations in Prydz Bay indicate that NO_3_ was not depleted even at the height of the open water bloom^10^, and the maximum winter NO_3_ was greater than 25 μmol kg^−1^, throughout the (well-mixed) water column^10^. The uptake of 6.5 μmol kg^−1^ ‘additional’ NO_3_ would require the input of 0.04 to 0.4 nmol L^−1^ Fe, based on the Fe:NO_3_ = 2.0 nM (μM)^−1^ phytoplankton uptake ratios for open and coastal Southern Ocean waters^34^,^35^. Such increases are well within the range of Fe supply by melting sea-ice^36^,^37^. Recent observations in the Mertz Polynya (East Antarctica) have shown that the input of melt water from glacial and sea-ice increased biological production, with an associated enhancement of surface Ω of five-times the expected decrease due to dilution^38^. Thus, in summer, Antarctic systems may buffer anthropogenic decreases in pH and Ω with increased biological production, wherever melting provides sufficient additional supply of Fe.

It is important to note that while the studies presented here represent rare annual coverage of the carbon system in both Arctic and Antarctic coastal systems, the data were collected over a period of 13 to 15 months and therefore do not allow any assessment of potentially large interannual variability. New observations from the Prydz Bay study site in the Antarctic indicate that interannual variability in hydrography and biological processes resulted in larger changes than those caused by the decadal scale increase in anthropogenic CO_2_ (ref. 16). Additional annual observations of physical and biogeochemical cycles in Polar Regions are clearly needed before the impacts of future changes in the climatically and chemically sensitive high-latitude regions can be properly evaluated. In particular, observations of the systems outside of the period of biological activity, and thus strong (seasonal) pH increases are essential. As this work clearly shows, the (natural) biological CO_2_ drawdown (and corresponding pH and Ω enhancement) in summer, are much larger than the anthropogenic CO_2_ increases (and resulting pH and Ω suppressions). Thus, if we rely on these summertime observations for projections of future changes, the baseline in these (weakly buffered) systems is represented by the least vulnerable CO_2_ system state. This point has been well illustrated in the Southern Ocean, where the inclusion of carbon system seasonality (i.e., minimum winter values of pH and Ω) in the assessment of ocean acidification brought forward the onset of carbonate undersaturation by several decades^39^.

## Methods

### CO_2_ system data – Amundsen Gulf

Dissolved inorganic carbon (DIC) and alkalinity (TA) data were collected in Amundsen Gulf (Canadian Arctic Archipelago, Fig. 1a) from September 2007 through July 2008 as part of the Canadian International Polar Year Circumpolar Flaw Lead (IPY-CFL) System Study^9^. DIC (±2 μmol/kg) and TA (±2 μmol/kg) analysis were made by coulometric, and potentiometric titration, respectively, using a VINDTA 3C (Marianda), and following standard procedures^40^. The computation of CO_2_ partial pressure (pCO_2_, ±3 μatm), pH (±0.01) and aragonite saturation state (Ω, ± 0.01), was via the CO_2_SYS program of Lewis and Wallace^41^ and the equilibrium constants of Dickson and Millero^42^.

### CO_2_ system data – Prydz Bay

DIC and pH data were collected from late December 1993 to mid-January 1995 in Prydz Bay (East Antarctica, Fig. 1b)^10^. DIC (±2 μmol/kg) was measured by coulometric titration using a SOMMA analyser at CSIRO in Hobart, Australia, calibrated against certified seawater standards^10^; pH (±0.01) was measured spectrophotometrically (in Prydz Bay) after warming samples to 25°C (ref. 10). The DIC and pH data were used to compute TA (±5 μmol/kg), pCO_2_ (±4 μatm), and Ω (±0.01) via the CO_2_SYS program as described above.

To account for the mismatch in sampling dates, the Prydz Bay data were projected to 2007 for comparison of the carbonate system in a common year. We assumed that the surface ocean tracked a (constant) atmospheric pCO_2_ increase of 1.7 ppm yr^−1^, which has been reported based on large scale observations of pCO_2_ in the Southern Ocean^13^, and at the long-term, open ocean Bermuda Atlantic Time Series (BATS) station^14^, and Hawaii Ocean Time Series (HOT) station^15^. The Prydz Bay data, collected in 1994, were thus scaled by an atmospheric pCO_2_ increase of 24 ppm (ΔDIC_PB,2010_ = 15 μmol kg^−1^) to the year 2007 assuming constant temperature, salinity and total alkalinity.

### Net community production computations

In both the Amundsen Gulf^9^ and Prydz Bay^10^, seasonal changes in the CO_2_ system are dominated by biological processes and the addition/removal of freshwater due to melting/formation of sea-ice, and in the case of Amundsen Gulf the addition of freshwater from river runoff. Changes in salinity-normalised DIC, (i.e., nDIC = 35DICobs/Sobs, where the subscript ‘obs’ refers to the observed or in situ value), were thus used to estimate changes in DIC due to biological process^4^, yielding an estimate of net community production (NCP, i.e., NCP = −ΔnDIC), neglecting the contribution from air-sea exchange of CO_2_, since seasonal CO_2_ uptake in both regions makes a negligible contribution to changes in water column DIC^9^,^10^. This approach assumes that resupply of DIC-rich subsurface or offshore water can be neglected. In Amundsen Gulf, Shadwick et al^9^. have shown that this contribution is small compared to the effects of freshwater and biological production. In Prydz Bay, the water column is shallow (20 m) and well mixed throughout the year^9^ Furthermore, the flow of the Prydz Bay is slow and we thus also neglect resupply in this region.

### Projections of CO_2_ parameters to the year 2100

The impact of anthropogenic CO_2_ uptake on both the Amundsen Gulf (AG) and Prydz Bay (PB) systems was investigated via projection of the seasonal cycles of the carbonate system to the year 2100. We extended the atmospheric pCO_2_ forward at a rate of 1.7 ppm yr^−1^ (ref. 13) to arrive at ~550 ppm CO_2_ in 2100. This is equivalent to the most optimistic (B1) IPCC scenario under which population stabilizes by mid-century and technological advances reduce emissions, and much less than the business as usual (A1) scenario which reaches 1000 ppm CO_2_ (ref. 27). The assumption that surface ocean pCO_2_ tracks the atmospheric increase is validated by broad scale observations of surface pCO_2_ spanning several decades^13^. Furthermore, as our objective is not to accurately predict future values, but to comment on the vulnerability to broadly expected changes in the system, our results are not particularly sensitive to the rate of surface ocean CO_2_ increase chosen.

For both projections we assumed that the seasonal cycles are unchanged, and that TA remains constant at mean winter values (i.e., TA_AG_ = 2220 μmol kg^−1^, and TA_PB_ = 2320 μmol kg^−1^). The Amundsen Gulf data, collected in 2007, were scaled by an atmospheric pCO_2_ increase of 158 ppm to the year 2100 (ΔDIC_AG,2100_ = 78 μmol kg^−1^). The Prydz Bay data, collected in 1994, were scaled by an atmospheric pCO_2_ increase of 180 ppm (ΔDIC_PB,2100_ = 70 μmol kg^−1^). In addition to anthropogenic CO_2_ loading, the impact of increased surface water temperature and decreased salinity on pH and Ω was investigated. Following the 2001 report of IPCC WG2 (ref. 43), we applied an increase to surface water temperature of 6°C in the Arctic and 3°C in the Antarctic, and a freshening of 0.1 units following the global trend in salinity of Boyer et al^44^. for the period between 1955 to 1998 (0.001 yr^−1^) to both regions, though this is certainly an underestimate for the high latitude oceans. These changes were applied uniformly to the observed monthly values, with an additional proportional decrease in DIC and TA for dilution by freshening.

The assumption that DIC and TA will evolve conservatively with salinity is a very simple approach that does not allow resolution of the complex interaction between freshwater from both sea-ice melt and riverine sources (riverine TA ranges from 1500 to 1800 μmol kg^−1^ in the Amundsen Gulf region^9^, and contributes less than 3% by volume to the upper 50 meters of the water column^45^). Importantly, the input of both riverine and sea-ice melt water contribute freshwater with lower alkalinity than the oceanic values, and so both dilute TA (though by different amounts), weakening the alkalinity buffering. Furthermore, a recent study using two versions of an Earth System Model assessed the sensitivity of changes in pH and Ω in the Arctic Ocean under two different atmospheric forcing scenarios^46^. They conclude that the reduction in pH and Ω by freshwater is larger than the increase due to (increased) biology, but both these drivers are an order of magnitude smaller than the changes caused by increased atmospheric CO_2_ uptake and subsequent storage^46^. These projections depend on model choice of CO_2_ system parameters, and of particular importance to the Arctic system, on the surface TA concentration, which varied by more than 50 μmol kg^−1^ between models^46^. We have not included any parameterization of potential (and likely) feedbacks, as done in the Southern Ocean, for example, by McNeil and Matear^39^, as our work was not intended to give a model projection of the values of pH and Ω expected in a future Arctic and Southern Ocean, rather to asses the vulnerability to change based on the only existing observations of the current seasonality. The assumption that the seasonal cycles in both regions remain unchanged is likely an oversimplification, however, given the paucity of observations resolving the seasonality in the carbon system in polar waters and the poor performance of climate models, particularly in the Arctic Ocean^47^, this exercise is particularly instructive in highlighting the contrasting sensitivities of the Arctic and Antarctic systems to anthropogenic change.

## Author Contributions

E.H.S. led the study. T.W.T., H.T. and J.A.E.G. contributed to the writing of the manuscript and the interpretation of results. All authors contributed to the analysis of observational data.

## Figures and Tables

**Table 1:** Arctic Summer Surface Properties. The Amundsen Gulf site is representative of coastal conditions observed elsewhere in the Western Arctic^17^, and Arctic Archipelago^18^, in regions dominated by cold, low-salinity surface waters. Regions like the Barents Sea^19^, dominated by warmer, more saline, Atlantic inflow, are not well represented by the Amundsen Gulf system. Units of Dissolved Inorganic Carbon (DIC) and Total Alkalinity (TA) are μmol kg^−1^

	Amundsen Gulf^9^	Chukchi Sea^17^	North Water^18^	Barents Sea^19^
**T**	0 < T < 8	0< T < 8	−0.3 < T < 2.6	2 < T < 6
**S**	<31	<32.5	<32.5	35.04
**DIC**	1950 to 2000	1900 to 2000	1910 to 2120	~2140
**TA**	2050 to 2150	2100 to 2200	2100 to 2230	~2300

**Table 2:** Antarctic Summer Surface Properties. The Prydz Bay site is representative of coastal conditions observed throughout the Antarctic region. In particular, the onset and duration of the productive season, and annual DIC and nitrate minima in the Ross Sea^20^,^21^ are similar to conditions observed in the shallower Prydz Bay system. The relatively warmer, and fresher Antarctic Peninsula^23^ is not well represented by the hydrographic and CO_2_ system parameters observed in Prydz Bay. Units of DIC and TA are μmol kg^−1^

	Prydz Bay^10^	Ross Sea^20^,^21^	Weddell Sea^22^	Antarctic Peninsula^23^
**T**	−1.8 < T < 0	−1.8 < T < −1.6	−1.8 < T < −1.0	0 to 2.5
**S**	33 < S < 34	~34	~34	33 < S < 33.6
**DIC**	1980 to 2050	2050 to 2150	2140 to 2220	2100 to 2150
**TA**	2250 to 2280	2260 to 2300	2280 to 2340	2250 to 2350

**Table 3:** Monthly estimates of NCP (mol C m^−2^) in Amundsen Gulf and Prydz Bay. Positive values indicate biological production and negative values indicate respiration, or remineralisation. In Amundsen Gulf, annual NCP amounts to roughly 1.33 mol C m^−2^ yr^−1^, which is exported to the subsurface layer^9^,^30^. In the shallower Prydz Bay, there is no annual accumulation of NCP, since organic matter produced in the spring and summer is respired in the autumn and winter seasons^10^

Month	NCP^AG^	Month	NCP^PB^
J	−0.39	J	−0.11
F	−0.10	A	−0.06
M	−0.26	S	−0.11
A	0.45	O	−0.03
M	0.81	N	0.09
J	1.11	D	0.26
J	1.02	J	1.36
A	0.28	F	0.64
S	−0.33	M	−0.28
O	−0.24	A	−0.74
N	−0.33	M	−0.74
D	−0.65	J	−0.25
Total	1.33		0.02
